# The Relationship Between Cognitive Status and Known Single Nucleotide Polymorphisms in Age-Related Macular Degeneration

**DOI:** 10.3389/fnagi.2020.586691

**Published:** 2020-10-16

**Authors:** Caitlin Murphy, Aaron P. Johnson, Robert K. Koenekoop, William Seiple, Olga Overbury

**Affiliations:** ^1^Low Vision Lab, School of Optometry, University of Montreal, Montreal, QC, Canada; ^2^Concordia Vision Labs, Department of Psychology, Concordia University, Montreal, QC, Canada; ^3^Centre for Interdisciplinary Research in Rehabilitation of Greater Montreal (CRIR)/Centre de Réadaptation Lethbridge-Layton-Mackay du Centre Intégré Universitaire de Santé et de Services Sociaux du Centre-Ouest-de-l’Ile-de-Montréal (CIUSSS) du Centre-Ouest-de-l’Île-de-Montréal, Montreal, QC, Canada; ^4^Paediatric Surgery and Human Genetics and Ophthalmology, Faculty of Medicine, McGill University Health Centre, Montreal QC, Canada; ^5^Arlene R. Gordon Research Institute, Lighthouse Guild, New York, NY, United States; ^6^School of Medicine, New York University, New York, NY, United States; ^7^Lady Davis Institute of Medical Research, Montreal, QC, Canada

**Keywords:** age-related macular degeneration, mild cognitive impairment, low vision, genetics, complement factor H, age-related maculopathy susceptibility gene 2, fatty acid desaturase 1

## Abstract

Recent literature has reported a higher occurrence of cognitive impairment among individuals with Age-related Macular Degeneration (AMD) compared to older adults with normal vision. This pilot study explored potential links between single nucleotide polymorphisms (SNPs) in AMD and cognitive status. Individuals with AMD (*N* = 21) and controls (*N* = 18) were genotyped for the SNPs CFHY402H, ARMS2A69S and FADS1 rs174547. Cognitive status was evaluated using the Montreal Cognitive Assessment. The two groups differed significantly on which subscales were most difficult. The control group had difficulty with delayed recall while those with AMD had difficulty on delayed recall in addition to abstraction and orientation. Homozygous carriers of the FADS1 rs174547 SNP had significantly lower scores than heterozygotes or non-carriers on the MoCA. The results suggest that the FADS1 SNP may play a role in visual impairment/cognitive impairment comorbidity as reflected in the poorer cognitive scores among homozygotes with AMD compared to those carrying only one, or no copies of the SNP.

## Introduction

With the aging of the population, the number of individuals affected by Age-related Macular Degeneration (AMD) is on the rise. AMD is presently the leading cause of legal blindness in industrialized nations, with a prevalence that increases with age (Klein et al., 2007). To date, AMD is understood to be a retinal degenerative condition that impairs, among others, the ability to read, to recognize faces, and to drive, all of which can lead to a decreased quality of life and loss of autonomy.

In addition to a higher prevalence of AMD, older adults (60 + years old) have a higher prevalence of cognitive impairment. Cognitive impairment refers to a decrease in a person’s ability to remember and think, to an extent that it interferes with the ability to perform daily activities. At present, the World Health Organization estimates that ∼ 5–7% of the population aged 60 and over have some type of cognitive impairment, with the most common type being Alzheimer’s disease (AD) (World Health Organization, 2012; Wortmann, 2012). There is a growing body of scientific literature linking both AMD and cognitive impairment. At the turn of the millennium, large-scale population-based studies began reporting a higher prevalence of cognitive impairment among individuals with AMD (Klaver et al., 1999; Wong et al., 2002). In the first decade of the 2000s, researchers determined the shared risk factors and histopathological characteristics (Terai et al., 2001; Johnson et al., 2002; Anderson et al., 2004; Katta et al., 2009). Most notably, beta-amyloid (βA), best known as a component of the senile plaques found in the brains of individuals with AD was also identified as a component of drusen, the hallmark deposits of AMD (Johnson et al., 2002). Further, beta-amyloid was found to form similar vesicular structures in senile plaques and drusen (Anderson et al., 2004).

With further advances in science and technology, and based upon the data released from genome-wide association studies (GWAS), researchers have refined their abilities to study complex diseases, i.e., diseases caused by a combination of genetic, environmental and lifestyle factors. Characterizing the contribution of a single factor to a complex disease is difficult due to it being obscured or confounded by other contributing factors (Craig, 2008). A reasonable place to start is the examination of the relationship between genetic factors and associated phenotype. Such associations can lead to better knowledge of disease mechanisms and to treatment options. AMD and AD are prime examples of complex diseases. Both have benefited from the information gained from GWAS but have, so far, been studied separately in terms of genetic factors and associated phenotypes.

The current pilot study investigated AMD-cognitive impairment comorbidities with respect to possible common genetic factors. Similar disease risk factors in AMD and AD and common histopathology lead to the hypothesis that gene mutations may result in common pathogenesis in AMD and AD. The first mutation to be associated with AMD was the Y402H single nucleotide polymorphism (SNP) in Complement Factor H (*CFH*). *CFH* is the gatekeeper for the complement cascade, and a mutation impairing its function results in increased inflammation. This association was reported by four studies in 2005 (Edwards et al., 2005; Hageman et al., 2005; Haines et al., 2005; Klein et al., 2005). Inflammation was first associated with AD in 1907 by Aloysius Alzheimer himself (Alzheimer et al., 1995). In addition, βA, a feature of both AMD and AD has been shown to trigger the complement cascade (Johnson et al., 2002). Considering that complement-driven inflammation and βA are implicated in both AMD and AD, the same polymorphisms that infer risk for AMD may also modulate AD risk.

A second SNP having a significant impact on AMD risk is Age-related Maculopathy Susceptibility gene 2 (*AMRS2*) A69S. Compared to *CFH*, *ARMS2* is not as well characterized. Research to date has found that it is expressed in both the brain and in the retina (Gatta et al., 2008). A recent study finding that *ARMS2* may be involved in complement-mediated clearance of cellular debris (Micklisch et al., 2017). The A69S SNP appears to cause mRNA instability, resulting in a deficiency of the protein. Without the *ARMS2* protein present, the complement cascade is not activated to clean up necrotic cells and unwanted debris. This can lead to the formation of drusen and senile plaques that are the characteristics observed in AMD and AD respectively.

The Fatty Acid Desaturase 1 (*FADS1*) SNP, rs174547, has also been identified as a contributing factor toward AMD through GWAS (Neale et al., 2010), but its role is even less well characterized. The *FADS1* gene encodes an enzyme involved in lipid metabolism, one of the pathogenic systems contributing to AMD and AD. More specifically, *FADS1* encodes delta-5 fatty acid desaturase, the rate-limiting enzyme required for polyunsaturated fatty acid (PUFA) biosynthesis. It is involved in the omega-3 and omega-6 pathways (Martinelli et al., 2008; Dumont et al., 2011). The rs174547 SNP causes an increase in enzyme activity, which is thought to contribute to AMD in two ways. First, omega-3 and omega-6 pathways compete for use of the delta-5 enzyme, with the omega-6 pathway coming out ahead. This means there is always a lower level of omega-3 PUFAs compared to omega-6. Due to this, there is less docosahexaenoic acid (DHA), a long-chain omega-3 fatty acid that accounts for 50% of the lipid content of photoreceptor rod outer segments (Cakiner-Egilmez, 2008; Augood et al., 2008; Tuo et al., 2009). Retinal function depends on DHA and deficiencies of omega-3 PUFAs have been shown to alter photoreceptor function (Cakiner-Egilmez, 2008). Secondly, omega-6 PUFAs compete with omega-3s for incorporation into cell membranes. The presence of omega-3s in cell membranes serves to dampen inflammatory response but, without them, the inflammation brought about by high levels of pro-inflammatory omega-6 PUFAs can go unchecked (Cakiner-Egilmez, 2008; Serini et al., 2011).

In terms of AD, the condition of the *FADS1* gene product is important for the structural integrity of the brain. Approximately half of the brain’s dry mass is composed of omega-3 PUFAs, the lipids that depend on *FADS1* for their biosynthesis, and ∼90% of this is DHA (Weiser et al., 2016). DHA is used in the phospholipid membranes of brain cells and also serves as a precursor for bioactive molecules required for brain function (Freemantle et al., 2012). It is enriched at synaptic terminals and changes in its concentration can affect cellular characteristics and physiological processes such as neurotransmitter release, signal transduction, neuroinflammation and neuronal differentiation and growth (Uauy and Dangour, 2006; Orr and Bazinet, 2008).

The current pilot study examined the frequency of the SNPs *CFHY402H*, *ARMS2A69S*, and *FADS1* rs174547, and cognitive status in individuals with AMD and controls. The SNPs were expected to occur more frequently in the AMD group compared to controls and in individuals scoring positive for mild cognitive impairment. Cognitive status was measured using the Montreal Cognitive Assessment (MoCA), which has been shown to be sensitive to mild cases of cognitive impairment (Dag et al., 2014).

## Materials and Methods

Participants were recruited from the Montreal Retina Institute and the School of Optometry Clinic at the Université de Montréal. The study protocol was approved by *Le Comité d’éthique de la recherche en santé at the Université de Montréal*, and followed the tenets of the Declaration of Helsinki. All study participants gave signed informed consent prior to their participation in the study.

Individuals aged 70 years or older and diagnosed with AMD by an ophthalmologist or optometrist were recruited for this study. The control group consisted of participants aged 70 years or older recruited through word of mouth. They were required to have normal vision as determined by Early Treatment of Diabetic Retinopathy visual acuity and healthy retinas as determined by optical coherence tomography. Those with comorbid glaucoma, neurological disorders, or a diagnosis of dementia were excluded.

For the 107 participants from the Montreal Retina Institute, genotyping was conducted as part of a previous study (Smailhodzic et al., 2012a) by Radboud University Medical Center in Nijmegen, Netherlands. The remaining patients and all control participants were genotyped through Sanger sequencing-based targeted mutation analysis by Asper Biogene Ltd. In Estonia. DNA was extracted from participate saliva samples. Presence of the Y402H SNP in *CFH* (rs1061170), the A69S SNP in *ARMS2* (rs104909245), and rs174547 SNP in *FADS1* was reported.

## Cognitive Assessment

The MoCA is designed to detect mild cognitive impairment. It screens several cognitive domains for a total score of 30 points. Individuals scoring less than 26 points are considered to have screened positive for mild cognitive impairment (Nasreddine et al., 2005). A blind version has been validated for use in visually impaired individuals (Wittich et al., 2010; Jefferis et al., 2012). The MoCA Blind omits questions requiring vision (e.g., copying a shape) and recalculates the total. It is scored out of 22, and considers a score below 18 points as screening positive for mild cognitive impairment. However, the removal of the visual components of these questionnaires reduces the overall sensitivity of the MoCA (Busse et al., 2002), leading to underestimation of scores. It is recommended to use the full version when possible, and interpret the score for all questions (full MoCA) and only the non-visual questions (MoCA Blind) separately (Busse et al., 2002).

## Analysis

Since the data were not normally distributed with a skewness of −0.810 (*SE* = 0.378) and kurtosis of 0.310 (*SE* = 0.741), the non-parametric Mann-Whitney *U*-test was used to compare ranks of the cognitive measures between and within the AMD group and the control group. The same test was also used to compare data between carriers and non-carriers of each SNP of interest. A Kruskal-Wallis non-parametric one-way ANOVA was used to compare the results of cognitive tests across zygosity for each SNP. To provide more information about the distribution of these data (which can be more informative than differences between mean/median; Rousselet et al., 2017), data were divided into deciles with 95% confidence intervals. The differences between deciles in the AMD vs. Control groups were calculated to identify any meaningful difference between groups. All calculations were conducted using SPSS software, version 20.0. and JASP version 0.8.1.2 (IBM Corporation, 2011; JASP Team T, 2017).

## Results

A total of 107 individuals were genotyped from a previous study (Smailhodzic et al., 2012b). From this sample, mortality or development of AD since genotyping excluded 15 potential participants, six were unreachable, two were excluded because they were under 70 years of age, and 74 declined further participation. This left a sample of 10 individuals (3M, 7F) who completed the test battery. An additional 11 participants were added to the AMD group for a total of 21 individuals (4M, 17F) with an average age of 78.9 years (range: 71–92) and average binocular visual acuity of 0.27 logMAR (range: −0.10–1.00 logMAR). The control group consisted of 18 individuals (6M, 12F) with an average age of 74.1 years (range: 70–85) and average visual acuity of −0.02 logMAR (range: −0.26 – 0.16 logMAR).

Genetic testing determined that there were 21 carriers of *CFHY402H*, with 50% of AMD and 9% of control participants being homozygous. There were also 21 carriers of *ARMS2A69S*, with 41.6% of AMD and 11% of control participants being homozygous. There were 33 carriers of the *FADS1* SNP with 53% of AMD and 37.5% of control participants being homozygous. The results of genetic testing are summarized in Table 1.

**TABLE 1:** Genetic results.

SNP	AMD	Control	Total
**CFHY402H** (rs1061170)	10	11	21
Homozygotes	5	2	6
**ARMS2A69S** (rs10490924)	12	9	21
Homozygotes	5	1	6
**FADS1** (rs174547)	17	16	33
Homozygotes	9	6	15

## Cognitive Questionnaires

**FIGURE 1:**
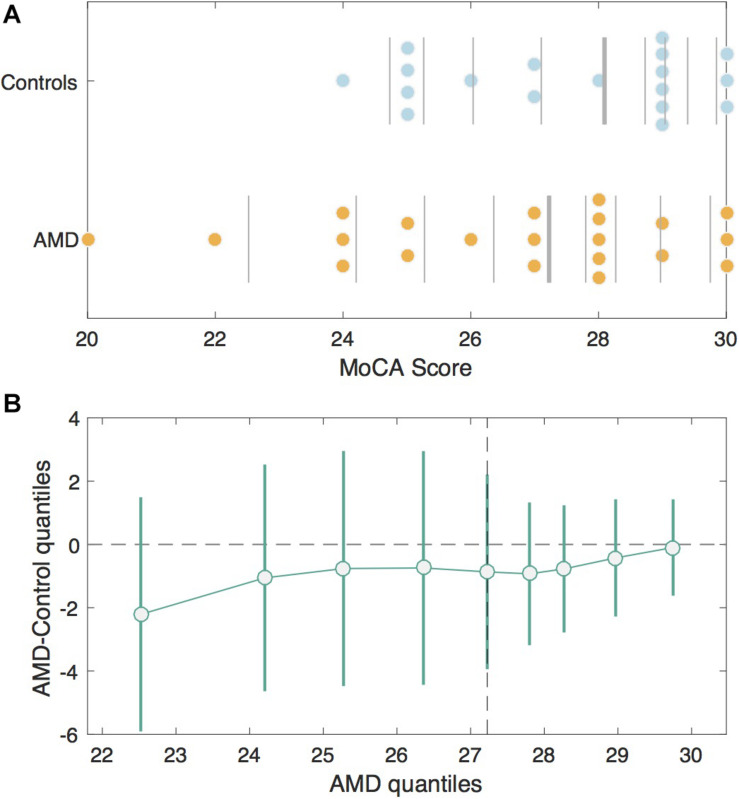
MoCA Scores in the AMD group v. Controls. **(A)** Jittered scatterplots of the MoCA scores for the Control group (blue) and the AMD group (orange). The vertical lines denote deciles for each group. The thickest vertical line in each group represents the median. **(B)** The shift function or differences in corresponding deciles between control and AMD groups with 95% bootstrapping confidence intervals. The deciles for the control group are plotted on the *x*-axis and the differences between control and AMD deciles are plotted on the *y*-axis. The difference is greatest for the first decile, since AMD participants had lower scores, but the confidence intervals cross zero, indicating this difference is not significant.

The MoCA scores were not significantly different between groups (AMD median = 27; Control median = 28.5), *U* = 150.00, *p* = 0.27 (Figure 1). Seven AMD participants (33.3%) and five control participants (27.8%) screened positive for mild cognitive impairment (MCI) according to the MoCA. When scores for the blind version of the MoCA were calculated, only one of the AMD subjects who screened positive for MCI in the original scoring achieved a score inside the normal range (≥18 out of 22).

Although the average MoCA scores did not differ between the AMD group and controls, the subscales they had difficulty with did. Those from the control group scoring positive for MCI had significantly lower scores on the delayed recall subscale compared to those from the same group who passed, *U* = 2.5, *p* = 0.002. Comparatively, those from the AMD group with MCI scored significantly poorer on delayed recall, *U* = 14.5, *p* = 0.005, in addition to the orientation, *U* = 37.5, *p* = 0.034, and abstraction, *U* = 24.5, *p* = 0.007, subscales of the MoCA compared to the rest of the AMD group. See Supplementary Material for details of the results of the cognitive questionnaires.

## Genetic Testing

The *CFHY402H* SNP was carried by seven of the 12 who scored below normal on the MoCA. Two of them were homozygotes, neither of which had AMD. Scores obtained by carriers and non-carriers of *CFHY402H* were not significantly different. There was no relationship between *CFH* zygosity and cognitive scores.

The *ARMS2* SNP was carried by five of the individuals who scored below the normal range on the MoCA. They were all heterozygous for the SNP, with four of them being from the AMD group and one from the control group. MoCA scores did not differ significantly between carriers and non-carriers of *ARMS2A69S*. There was no relationship between *ARMS2* zygosity and cognitive scores.

**FIGURE 2:**
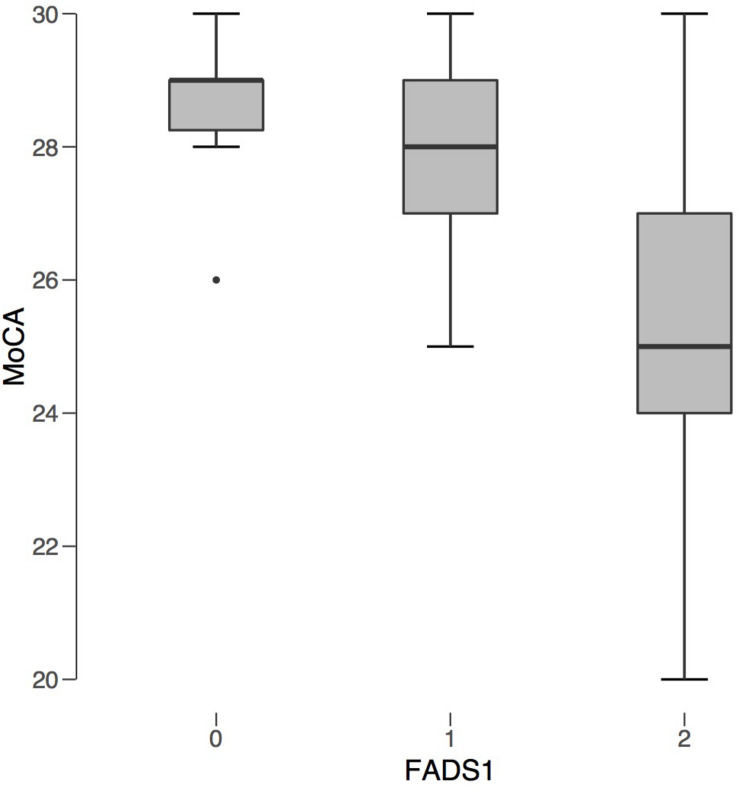
*FADS1* rs174547 Carrier Status v. Average MoCA Score. *FADS1* zygosity is plotted along the *x*-axis: 0 (non-carrier), 1 (heterozygous), 2 (homozygous). Average MoCA score is plotted on the *y*-axis. Regardless of AMD status, individuals homozygous for *FADS1* SNP rs174547 had significantly lower MoCA scores compared to non-carriers.

All 12 of those scoring positive for cognitive impairment on the MoCA were carriers of the *FADS1* SNP. Six of the seven from the AMD group with MCI were homozygotes, while three of the five from the control group were homozygotes. The proportion of homozygotes did not differ significantly between groups, χ^2^(2) = 1.26, *p* = 0.53. Kruskal-Wallis showed that homozygous carriers of the *FADS1* SNP had lower cognitive scores compared to heterozygous carriers and non-carriers, *H* = 8.52, *p* = 0.014, ε^2^ = 0.224 (Figure 2). *FADS1* SNP homozygotes with AMD had particular difficulty on the language and abstraction subscales.

## Discussion

A number of studies have reported an association between AMD and cognitive impairment (Klaver et al., 1999; Wong et al., 2002; Areds Research Group, 2006; Pham et al., 2006; Baker et al., 2009; Guo et al., 2010; Whitson et al., 2010; Ohno-Matsui, 2011; Kaarniranta et al., 2011; Whitson et al., 2011; Woo et al., 2012; Proitsi et al., 2012; Jonna et al., 2013; Keenan et al., 2014; Rozzini et al., 2014; Chung et al., 2015; Demirci et al., 2015; Harrabi et al., 2015; Tsai et al., 2015; Zhou et al., 2016). However, these studies have been limited regarding the evaluation of cognitive impairment. While some studies have used subscales of neuropsychological tests, such as the Wechsler Adults Intelligence Scale to assess cognitive function (Areds Research Group, 2006), these subscales cannot be used to reach a clinical diagnosis of cognitive impairment. Other studies have used the MMSE and found an association between cognitive impairment and late AMD, but not early AMD (Baker et al., 2009). There is evidence that the MMSE is not as sensitive to MCI (Hoops et al., 2009; Dag et al., 2014). As a result, it is possible that earlier stages of AMD could be associated with milder cognitive impairment that is too subtle to be detected by the MMSE, but which could be detected by the MoCA.

The percentage of AMD participants in this study who screened positive for MCI according to the MoCA (33%) is in agreement with previous studies (Duponsel et al., 2010; Wittich et al., 2014). The percentage of controls screening positive for MCI on the MoCA (27.8%) was high compared to MCI prevalence reported elsewhere in older adults (Lopez et al., 2003; Gauthier et al., 2006). Four of these five control subjects scored just under the standard cut off value of 26 (on the full MoCA).

Overall full (out of 30) and blind (out of 22) MoCA scores were not significantly different between the AMD group and the control group. However, the groups did differ on which subscales of the MoCA were difficult for participants. Those in the control group who screened positive for MCI on the MoCA had difficulty with delayed recall, which is typical of an MCI diagnosis (Rozzini et al., 2007; Summers and Saunders, 2012). Not all cases of MCI progress to AD. Prospective research has shown that cases of MCI presenting with deficits in memory in addition to deficits in other cognitive domains are more likely to convert to AD (Rozzini et al., 2007; Summers and Saunders, 2012). Those with AMD who screened positive for MCI on the MoCA had difficulty on the delayed recall subscale, but in addition also had difficulty with the orientation and abstraction subscores. This leads to the hypothesis that those with AMD screening positive for MCI on the MoCA may be at a higher risk of developing AD compared to controls. Prospective studies would have to be conducted to confirm this.

A greater number of women are affected by cognitive impairment and AMD compared to men. Our results agree with this. Originally this was thought to be due to differences in life span between men and women, but recent research has shown the relationship to be more complex than that. The biological role of sex in cognitive impairment, particularly AD, and AMD is largely understudied. Recent literature has reported exaggerated aging, a faster decline from MCI to AD, and a greater effect of amyloid pathology in females compared to males (Zhao et al., 2016; Sohn et al., 2018). If amyloid pathology has a greater effect on women compared to men, then both AD and AMD would be exacerbated in women.

The number of homozygous carriers of *CFH* and *ARMS2* SNPs was more than double in the AMD group compared to the control group. This is expected as presence of the *CFH* and *ARMS2* SNPS are said to account for over 50% of AMD cases (Haines et al., 2005; Haines et al., 2006). Although not statistically significant, homozygosity of the *FADS1* SNP was more frequent in the AMD group compared to controls. This agrees with numerous studies reporting the contributions of these SNPs to AMD (Edwards et al., 2005; Hageman et al., 2005; Jakobsdottir et al., 2005; Klein et al., 2005; Haines et al., 2006; Fritsche et al., 2008; Neale et al., 2010). However, *FADS1* rs174547 was present in the majority of the sample. To rule out sampling error, the carrier status of *FADS1* rs174547 in the original 107 was analyzed.

In the original sample, 91 individuals (85%) were found to be carriers and of them, 53.9% were homozygous. The alleles present at rs174547 are C, the ancestral allele, or T. The T allele is considered the risk allele for AMD. The major allele at this location differs depending on the population. The population of the current study, consisting mostly of individuals of French-Canadian heritage, could be considered most similar to a European or an American population. According to the dbSNP, a database of genetic and epidemiological information on SNPs from the National Institute of Health, the frequency for the T allele of rs174547 in an American population (0.41) is lower than that of a European population, at 0.65 (NIH, 2017). The frequency of the T allele in the current study is 0.85, greater than either the European or American frequencies. One explanation of this could be the Quebec Founder population effect (Roy-Gagnon et al., 2011). A Founder population is a new population that is established from few individuals (or founders) and, as a result, exhibits reduced genetic variation. Due to this, rare disease alleles are enriched, leading to higher numbers of homozygotes displaying the disease phenotype (Kristiansson et al., 2008). Such populations have been instrumental in medical genetics for research on genetic diseases. The Quebec population has been valuable in the study of genotype-phenotype interactions in Usher syndrome (Ebermann et al., 2009), and retinitis pigmentosa (Koenekoop et al., 2003; Coussa et al., 2015). The Founder Effect could potentially explain the increased frequency of the T allele at rs174547 in this study population.

In addition to occurring with greater frequency, the *FADS1* SNP was carried by all participants scoring positive for MCI on the MoCA. Homozygotes had the lowest cognitive scores, suggesting the *FADS1* SNP has a greater contribution to cognition than vision. The *CFHY402H* and *ARMS2A69S* SNPs appeared not to have an association with the results of cognitive questionnaires, as MoCA scores were not significantly different between carriers and non-carriers. This finding supports the biochemical research on *FADS1* discussed in the introduction. The presence of the rs174547 SNP increases delta-5 desaturase activity which, in turn, reduces DHA (Cakiner-Egilmez, 2008; Fauser et al., 2011; Merino et al., 2011; Hellstrand et al., 2012), a vital component for brain structure and cognition.

The Salisbury Eye Evaluation Study (Zheng et al., 2018) established a correlation between deterioration of vision and cognitive decline over an 8-year period. They also determined that the vision problems preceded cognitive decline, however, a causal relationship was not defined. The correlational relationship led to the sensory deprivation hypothesis, which states that a prolonged decline in sensory input will lead to cognitive decline due to neuronal atrophy (Valentijn et al., 2005; Clay et al., 2009). However, there is evidence to show to that vision impairment does lead to reorganization in the brain (Chen et al., 2019), but this is with respect to the processing of visual information, not memory and/or cognition. The few studies that have explored this hypothesis have not found results to support it (Hall et al., 2005; Anstey et al., 2006). Individuals with vision impairment often suffer from social isolation and depression (Renaud and Bédard, 2013; Zheng et al., 2018), both of which are also associated with cognitive decline (Potter and Steffens, 2007; Morimoto et al., 2015; Mick et al., 2017). It is important to consider that the relationship between vision and cognitive health could be mediated by these factors. Presently, there has been more evidence to support the common cause hypothesis, which considers aging to affect the physiology of the brain, causing decline in sensory and cognitive functions (Lindenberger and Baltes, 1994). The present study lends further support to this hypothesis by highlighting the potential role of the FADS1 SNP in age-related vision and cognitive decline.

This study is not without limitation, the most obvious being sample size. Since there has been little study on these SNPs in relation to cognitive impairment, there is no established minor allele frequency for cognitive impairment cases, which is required to determine appropriate sample size. Further, the current dataset is non-normally distributed – which means that a sensitivity analysis using g^∗^power is also not possible. It is hoped the results presented here will be the impetus to support larger scale studies in the future.

## Conclusion

Although the prevalence of MCI among participants with AMD was not much higher than controls in this sample, the prevalence is higher than that reported in other normally-sighted populations (Gauthier et al., 2006). Additionally, those with AMD scoring positive for MCI according to the MoCA had difficulty with different cognitive domains compared to controls scoring positive for MCI. This distribution of cognitive impairment indicates that those with AMD and MCI may be more likely to progress to AD than controls with MCI.

No significant associations between the most prominent AMD SNPs, *CFHY402H* and *ARMS2A69S*, and MCI were identified. This gives support to previous claims that although AMD and AD have many similarities, the underlying genetic mechanisms may be different (Proitsi et al., 2012). However, the findings were different for the *FADS1* SNP. Carriers, both with and without AMD, were more likely to have lower cognitive scores compared to non-carriers. Further, all those scoring positive for MCI according to the MoCA were homozygous for the *FADS1* SNP. These findings highlight the importance of testing for not only the prominent AMD SNPs but also the *FADS1* mutation in future studies.

Genetic studies of complex disease have recently become possible, but they have required vast study cohorts for an individual trait and international collaborations on enormous scales (Consortium, 2007). Large global populations may not always be necessary to study the genetics of complex diseases, like AMD and cognitive impairment. Susceptibility to complex disease involves contributions from common variants and rare variants. Several common variants are likely to explain a substantial fraction of the genetic contribution to a complex disease, while more rare variants have a greater impact on the phenotype of the disease. The statistical power required to detect susceptibility alleles is positively correlated with the frequency of the allele and the penetrance, or degree of phenotypic expression of the allele in the test population. Founder populations may be required to better define a risk allele, like *FADS1* rs174547 that, although significant, gets lost in GWAS as a result of population-specific effects. A number of researchers have discussed the advantages of using Founder populations in medical genetics. Some of the benefits include genetic, environmental and phenotypic homogeneity, good genealogical records, higher degree of linkage disequilibrium, and reduced allelic heterogeneity (Lohmueller et al., 2003; Cohen et al., 2004; Zeggini et al., 2005; Kristiansson et al., 2008). The results of this study suggest *FADS1* rs174547 may be a new focus for better understanding any common genetic mechanism in the AMD-MCI co-morbidity.

## Data Availability Statement

The datasets generated for this study can be found in online repositories. The names of the repository/repositories and accession number(s) can be found below: https://osf.io/zt26y/?view_only=4993bebe8e20457680fa72f5ee49934c, zt26y.

## Ethics Statement

The studies involving human participants were reviewed and approved by the Le Comité d’Éthique de la Recherche en Santé at the Université de Montréal. The patients/participants provided their written informed consent to participate in this study.

## Author Contributions

All authors contributed to study design, manuscript revision and have read and approved the final submitted version.

## Conflict of Interest

The authors declare that the research was conducted in the absence of any commercial or financial relationships that could be construed as a potential conflict of interest.
