# Supplementary material for: The Relationship Between Cognitive Status and Known Single Nucleotide Polymorphisms in Age-Related Macular Degeneration
Source: Front Aging Neurosci. 2020 Oct 16;12:586691. doi: 10.3389/fnagi.2020.586691 (PMC7596199; doi:10.3389/fnagi.2020.586691)
Supplement: Supplementary file 1 [file Table_1.DOCX]

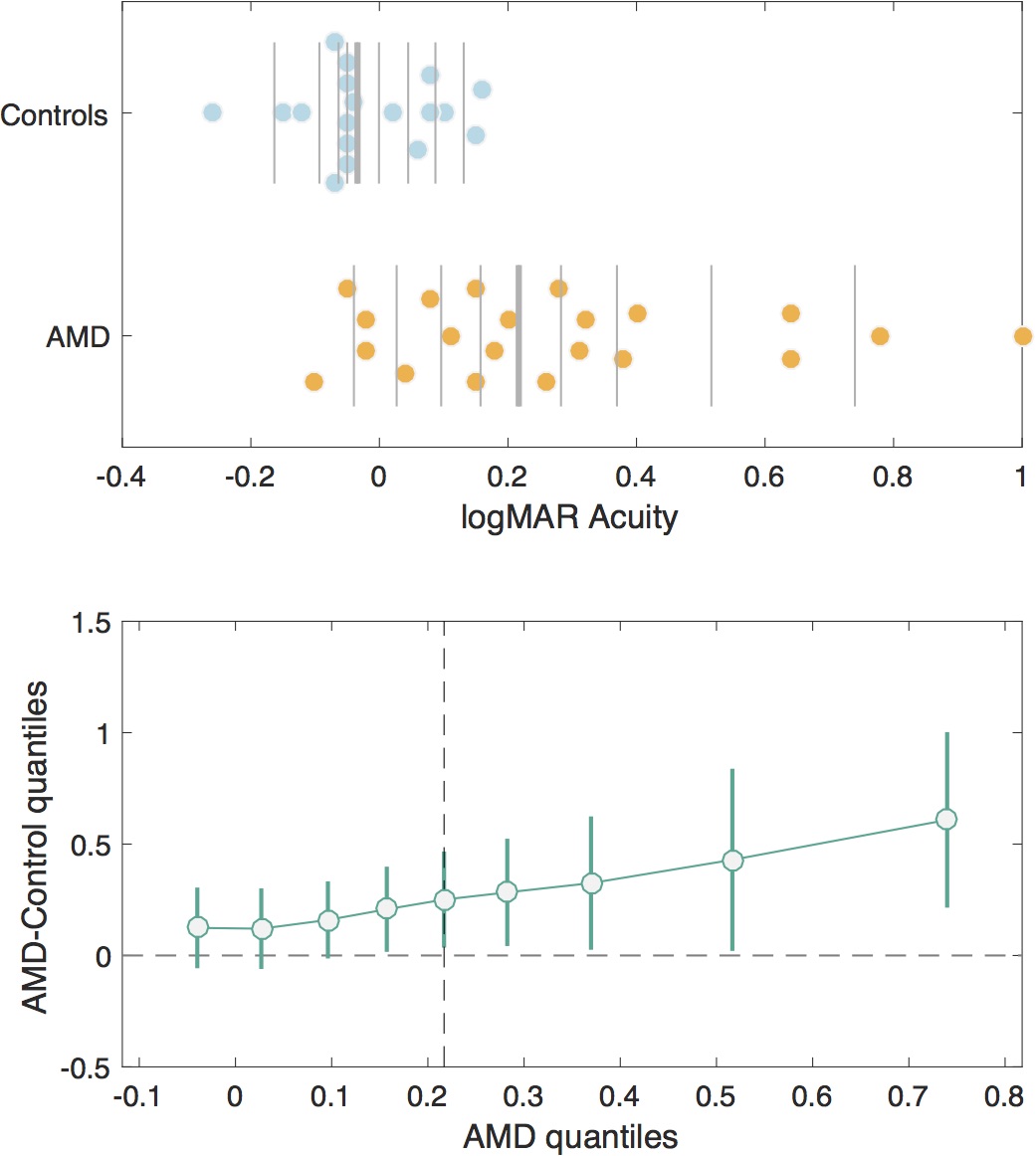


**Figure 1**. Visual Acuity in AMD group v. Controls

**A**. Jittered scatterplots of the logMAR visual acuities of the Control group (blue) and AMD Group (orange). The vertical lines denote the deciles for each group. The thickest vertical line in each group represents the median. **B**. The shift function calculates the differences in corresponding deciles between control and AMD groups with 95% bootstrapping confidence intervals. The deciles for the control group are plotted on the *x*-axis and the differences between control and AMD deciles are plotted on the *y*-axis. Deciles 1 to 3 have 95% confidence intervals that overlap 0, and thus are not significantly different. However, Decile 4 and higher, have confidence intervals that do no cross zero, indicating a significant difference between deciles with an alpha threshold of 0.05. This illustrates how much the AMD visual acuity distribution needs to be shifted to match the control group.
