# Supplementary material for: The Relationship Between Cognitive Status and Known Single Nucleotide Polymorphisms in Age-Related Macular Degeneration
Source: Front Aging Neurosci. 2020 Oct 16;12:586691. doi: 10.3389/fnagi.2020.586691 (PMC7596199; doi:10.3389/fnagi.2020.586691)
Supplement: Supplementary file 2 [file Table_2.DOCX]

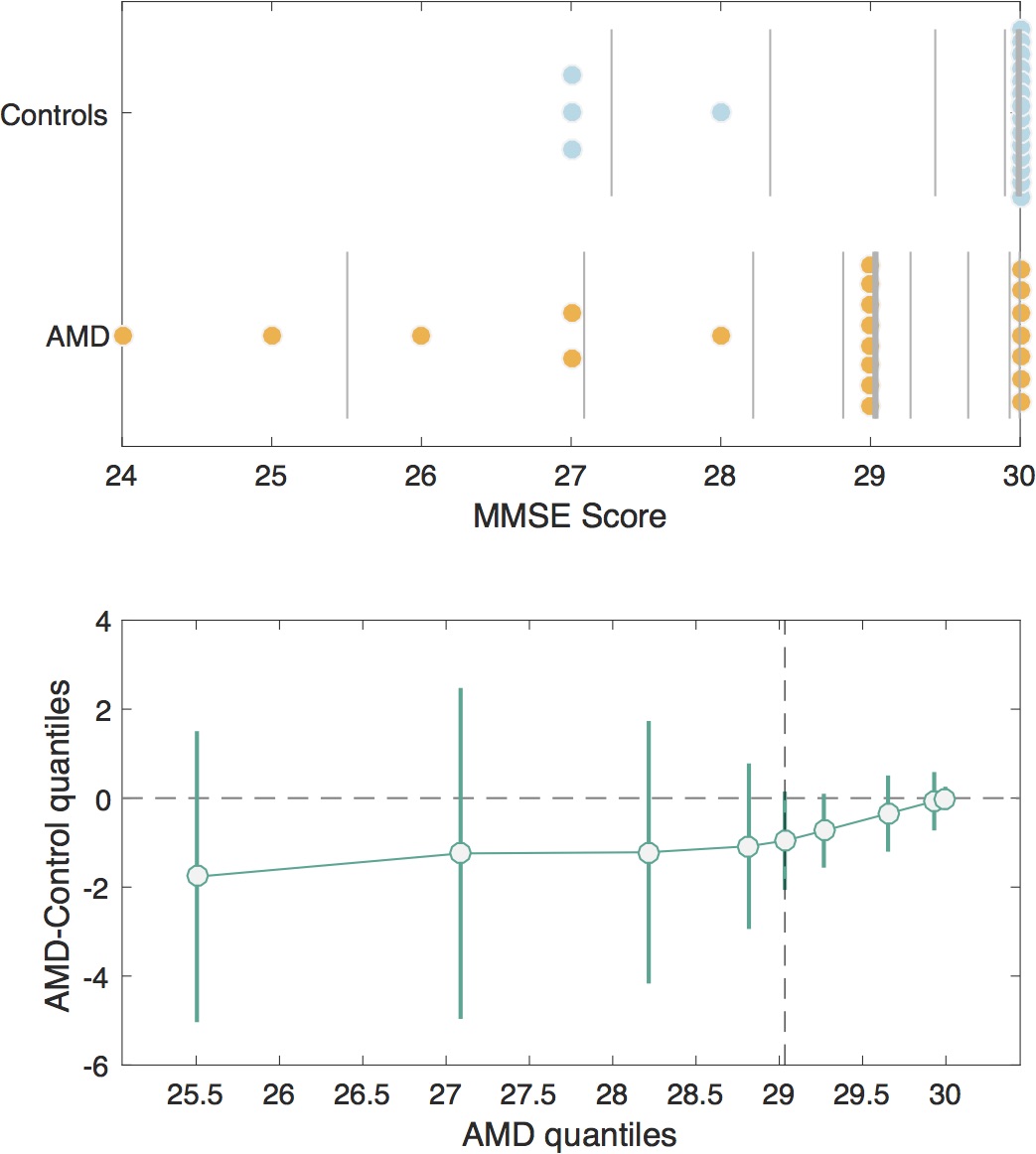


**Supplementary Figure 2**. MMSE Scores in the AMD Group v. Controls.

**A**. Jittered scatter plots of MMSE scores from the Control Group (blue) and the AMD group (orange). The vertical lines denote deciles for each group. The thickest vertical line represents the median. **B**. The shift function or differences in corresponding deciles between control and AMD groups with 95% bootstrapping confidence intervals. The deciles for the control group are plotted on the *x*-axis and the differences between the control and AMD deciles are plotted on the *y*-axis. Differences are negative, reflecting the lower scores from the AMD group. The confidence intervals cross zero for each decile, indicating there are no significant differences between the Control and AMD groups.
