# Supplementary material for: The Relationship Between Cognitive Status and Known Single Nucleotide Polymorphisms in Age-Related Macular Degeneration
Source: Front Aging Neurosci. 2020 Oct 16;12:586691. doi: 10.3389/fnagi.2020.586691 (PMC7596199; doi:10.3389/fnagi.2020.586691)
Supplement: Supplementary file 3 [file Table_3.DOCX]

**Supplementary Table I.** Cognitive Questionnaire Results: AMD Group

| **AMD Group** | | | | | | | | | | | |
| --- | --- | --- | --- | --- | --- | --- | --- | --- | --- | --- | --- |
| Age | VA | MoCA | MoCA Blind | MMSE | MM | CFHY402H | | ARMS2A69S | | FADS1 rs174547 | |
|  |  |  |  |  | Blind |  |  |  |  |  |  |
| 71 | -0.1 | 30.00 | 22.00 | 30.00 | 22.00 |  |  |  |  |  |  |
| 75 | 0.04 | 30.00 | 22.00 | 30.00 | 22.00 |  |  |  |  |  |  |
| 79 | -0.02 | 30.00 | 22.00 | 30.00 | 22.00 |  |  |  |  |  |  |
| 71 | 0.64 | 29.00 | 21.00 | 30.00 | 22.00 |  |  |  |  |  |  |
| 75 | 0.2 | 29.00 | 22.00 | 30.00 | 22.00 |  |  |  |  |  |  |
| 75 | 0.15 | 28.00 | 20.00 | 29.00 | 21.00 |  |  |  |  |  |  |
| 79 | 0.15 | 28.00 | 20.00 | 29.00 | 21.00 |  |  |  |  |  |  |
| 87 | -0.02 | 28.00 | 21.00 | 30.00 | 22.00 |  |  |  |  |  |  |
| 73 | -0.05 | 28.00 | 22.00 | 29.00 | 21.00 |  |  |  |  |  |  |
| 80 | 0.78 | 28.00 | 22.00 | 29.00 | 22.00 |  |  |  |  |  |  |
| 75 | 0.32 | 27.00 | 19.00 | 26.00 | 16.00 |  |  |  |  |  |  |
| 80 | 0.4 | 27.00 | 19.00 | 30.00 | 22.00 |  |  |  |  |  |  |
| 84 | 0.31 | 27.00 | 20.00 | 27.00 | 19.00 |  |  |  |  |  |  |
| 79 | 1 | 26.00 | 18.00 | 29.00 | 21.00 |  |  |  |  |  |  |
| 82 | 0.38 | 25.00 | 17.00 | 28.00 | 20.00 |  |  |  |  |  |  |
| 92 | 0.28 | 25.00 | 18.00 | 25.00 | 13.00 |  |  |  |  |  |  |
| 77 | 0.08 | 24.00 | 16.00 | 27.00 | 20.00 |  |  |  |  |  |  |
| 72 | 0.26 | 24.00 | 16.00 | 29.00 | 22.00 |  |  |  |  |  |  |
| 86 | 0.11 | 24.00 | 17.00 | 29.00 | 21.00 |  |  |  |  |  |  |
| 81 | 0.18 | 22.00 | 16.00 | 29.00 | 21.00 |  |  |  |  |  |  |
| 85 | 0.64 | 20.00 | 14.00 | 24.00 | 14.00 |  |  |  |  |  |  |
|  |  |  |  |  |  |  |  |  |  |  |  |
|  |  |  | SNP Key | Homozygous | |  |  |  |  |  |  |
|  |  |  |  | Heterozygous | |  |  |  |  |  |  |
|  |  |  |  | Non-carrier | |  |  |  |  |  |  |

Note: Visual acuity is recorded as logMAR. The MoCA is scored out of 30, with scores below 26 considered in the range of MCI. The MoCA Blind is scored out of 22 with scores below 18 considered in the range of MCI. The MMSE is scored out of 30 with scores below 25 considered in the range of cognitive impairment and the blind version is scored out 22 with 18-19 being the cut-off for those under 84 and 16-17 for those over 84 (Busse, Sonntag, Bischkopf, Matschinger, & Angermeyer, 2002).
